# Extended Near-Infrared Photoactivity of Bi_6_Fe_1.9_Co_0.1_Ti_3_O_18_ by Upconversion Nanoparticles

**DOI:** 10.3390/nano8070534

**Published:** 2018-07-16

**Authors:** Wen Ge, Zhiang Li, Tong Chen, Min Liu, Yalin Lu

**Affiliations:** 1 CAS Key Laboratory of Materials for Energy Conversion, Department of Materials Science and Engineering, University of Science and Technology of China, Hefei 230026, China; gew1024@mail.ustc.edu.cn (W.G.); lza0412@mail.ustc.edu.cn (Z.L.); aqct@mail.ustc.edu.cn (T.C.); 2 Key Laboratory of Advanced Technique & Preparation for Renewable Energy Materials, Department of Energy and Environmental Science, Ministry of Education, Yunnan Normal University, Kunming 650500, China; 3 Hefei National Laboratory for Physical Sciences at the Microscale, University of Science and Technology of China, Hefei 230026, China

**Keywords:** aurivillius, nanohybrid, upconversion, photocatalysis

## Abstract

Bi_6_Fe_1.9_Co_0.1_Ti_3_O_18_ (BFCTO)/NaGdF_4_:Yb^3+^, Er^3+^ (NGF) nanohybrids were successively synthesized by the hydrothermal process followed by anassembly method, and BFCTO-1.0/NGF nanosheets, BFCTO-1.5/NGF nanoplates and BFCTO-2.0/NGF truncated tetragonal bipyramids were obtained when 1.0, 1.5 and 2.0 M NaOH were adopted, respectively. Under the irradiation of 980 nm light, all the BFCTO samples exhibited no activity in degrading Rhodamine B (RhB). In contrast, with the loading of NGF upconversion nanoparticles, all the BFCTO/NGF samples exhibited extended near-infrared photoactivity, with BFCTO-1.5/NGF showing the best photocatalytic activity, which could be attributed to the effect of {001} and {117} crystal facets with the optimal ratio. In addition, the ferromagnetic properties of the BFCTO/NGF samples indicated their potential as novel, recyclable and efficient near-infrared (NIR) light-driven photocatalysts.

## 1. Introduction

Environmental contamination, especially water and air pollution, has become a serious problem, and thus has attracted great attention all over the world. Photocatalysisis known as an effective and promising technology in solving this problem [1,2,3,4,5,6,7]. In addition, photocatalytic materials have been widely used in membrane technology [8] and 2D materials [9,10]. Layered bismuth-based materials, namely BiFeO_3_ [11], BiVO_4_ [12], Bi_2_WO_6_ [13], Bi_2_MoO_6_ [14] and BiOX (X = I, Br, Cl) [15], have harvested special interest due to their unique electronic structure and decent photocatalytic properties. In particular, the orderly layered structure—composed of (Bi_2_O_2_)^2+^ fluorite-structure layers and perovskite-like or interleaved halide ions—is prone to form an internal electric field, which could efficiently separate the electron-hole pairs and result in enhanced photocatalytic activity [16,17].

Recently, layered bismuth-based Bi_4_Bi*_n_*_−3_Fe*_n_*_−3−*x*_Co*_x_*Ti_3_O_3*n*+3_ (BFCTO) Aurivillius compounds, which are formed by perovskite-type (Bi*_n_*_−1_Fe*_n_*_−3−*x*_Co*_x_*Ti_3_O_3*n*+1_)^2−^ blocks sandwiched between fluorite-type (Bi_2_O_2_)^2+^ slabs, where *n* is the number of perovskite units per half-cell [18,19,20,21], proved to be apotential single-phase multiferroic material with ferroelectricity and ferromagnetism [22]. Moreover, the internal electric field and ferromagnetism could enable the photocatalysts recyclable in viscous solution, which is important but extremely difficult due to their size and high viscosity [23]. In 2008, nanostructured Bi_5_FeTi_3_O_15_ was first realized, and showed excellent visible light-driven photocatalytic activity compared to bulk-Bi_5_FeTi_3_O_15_ and the widely-used photocatalyst TiO_2_ [24]. In 2014, Mandal et al. [25] successfully synthesized La^3+^ doped Bi_5_Ti_3_FeO_15_ (n = 4) via the solid-state reaction, and found that such layered oxides exhibited efficient photocatalysis for Rhodamine B (RhB) degradation under sunlight irradiation. In a previous study, the authors [26] optimized the photocatalysis in ferromagnetic Bi_6_Fe_1.9_Co_0.1_Ti_3_O_18_ (n = 5) nanocrystals by morphology control, and found that the synergistic effect of {001} and {117} facets in the separation of electrons and holes, and the oxidation/reduction reaction, efficiently inhibited the recombination of the charge carrier. Furthermore, we found the appropriate area ratio between the {001} facets and the {117} facets was shown to be beneficial to photocatalysis efficiency. Recently, we also obtained visible light-responsive Bi_7_Fe_3_Ti_3_O_21_ nanoshelf photocatalysts [27]. However, BFCTO photocatalysts are still limited to ultraviolet (UV) and/or visible (Vis) light, while near-infrared (NIR) light driven BFCTO-based catalysts have never been reported, despite NIR light making up about 47% of the solar spectrum. Therefore, the extended utilization of sunlight from Vis to NIR light is an attractive issue for developing photocatalysts. On the other hand, upconversion materials could convert low-energy NIR light into high-energy UV or Vis light through a nonlinear optical process [28,29,30,31,32,33]. Furthermore, compared with conventional fluorescent materials, such as organic dyes and quantum dots, upconversion nanoparticles have the following advantages: (i) High photostability and chemical stability; (ii) low toxicity to the human body; (iii) good biocompatibility by surface modification and functionalization; and (iv) good optical transparency over a wide wavelength range and low phonon energy [34,35,36,37]. Therein, coupling upconversion nanoparticles with photocatalysts is considered to bea very promising method to extend NIR photoactivity [38].

In this article, we synthesized firstly Bi_6_Fe_1.9_Co_0.1_Ti_3_O_18_ (BFCTO)/NaGdF_4_:Yb^3+^, Er^3+^ (NGF) nanohybrids by the hydrothermal process, followed by an assembly method, where the morphology and size of the BFCTO component was controlled by adjusting NaOH concentration. The structures, morphologies, upconversion emission, UV-Vis-NIR diffuse reflectance spectra and magnetic properties of the samples were characterized, and the photocatalytic activities were also evaluated by the degradation of RhB solution under the irradiation of light of wavelength 980 nm. The loading of the NGF upconversion nanoparticles extended the near-infrared photoactivity of all the BFCTO samples. BFCTO-1.5/NGF exhibited the best photocatalytic activity, and thus the corresponding mechanism was investigated in detail.

## 2. Experimental Section

### 2.1. Materials

All chemicals were purchased from Sinopharm Chemical Reagent Co., Ltd., (Shanghai, China), without further purification. The details are as follows: Ti(OC_4_H_9_)_4_ (≥99.7%), Bi(NO_3_)_3_·5H_2_O (≥99%), Fe(NO_3_)_3_·9H_2_O (≥98.5%), Co(NO_3_)_3_·6H_2_O (≥98.5%), HNO_3_ (65.0~68.0%), NaOH (≥96.0%), Gd(NO_3_)_3_∙6H_2_O (99.99%), Yb(NO_3_)_3_∙6H_2_O (99.99%), Er(NO_3_)_3_∙5H_2_O (99.99%), ethanol (99.7%), *cis*-oleic acid, NH_4_F (≥96.0%), HCl (36.0~38.0%), PVP (Polyvinylpyrrolidone, K-30).

### 2.2. Synthesis of BFCTONanoparticles with Various Morphologies

The BFCTO nanoparticles were synthesized by the hydrothermal method according to our previous report [23], and the corresponding morphologies were controlled by adjusting NaOH concentrations. Here, BFCTO-1.0, BFCTO-1.5 and BFCTO-2.0 represent BFCTO samples obtained with 1.0 M, 1.5 M and 2.0 M NaOH, respectively.

### 2.3. Preparation of PVP-Capped BFCTO Nanoparticles

The as-prepared BFCTO nanoparticles were added into the PVP solution (1.0 g PVP dissolved in 100 mL deionized water), and stirred for 12 h at room temperature. The obtained samples were isolated by centrifugation, washed several times with deionized water, and finally dried at 80 °C under vacuum for 12 h.

### 2.4. Synthesis of Oleic-Capped and Free NGF Nanoparticles

The oleic-capped NGF nanoparticles were synthesized using the solvothermal method. Firstly, 10 mL oleic acid and 3 mL NaOH solution (5 M) were successively added into 10 mL ethanol. After stirring for 10 min, 3.12 mL Gd(NO_3_)_3_, 0.80 mL Yb(NO_3_)_3_, 0.08 mL Er(NO_3_)_3_ and 2 mL NH_4_F (2 M) were added. Then the solution was transferred into a 50-mL Teflon-lined stainless-steel autoclave. Theautoclave was sealed and heated at 200 °C for 3 h and then cooled to room temperature. The obtained nanoparticles were collected by centrifugation, and then washed with water and ethanol for several cycles. Then the as-prepared oleic acid-capped NGF nanoparticles were dispersed in HCl solution (0.1 M) and ultrasonicated for 15 min to remove the ligands from the surface of the nanoparticles. The obtained NGF samples were collected via centrifugation and further purified by ethanol and deionized water for several rounds, and finally re-dispersed in deionized water.

### 2.5. Synthesis of BFCTO/NGF Samples

The as-prepared 0.3 g PVP-capped BFCTO nanoparticles were dissolved in 100 mL H_2_O, and 10 mL NGF solution (0.01 g mL^−1^) was added. Then, the solution was stirred for 12 h. The obtained nanoparticles were collected by centrifugation and washed several times with deionized water, and dried at 80 °C under vacuum for 12 h. 

### 2.6. Photocatalytic Activities

Photocatalytic activities of the as-prepared BFCTO/NGF samples were evaluated by the photodecomposition of RhB under the NIR irradiation of 980 nm (power = 1.0 A). Firstly, 50 mg of BFCTO/NGF and the BFCTO sampleswere dispersed uniformly into a 50-mL RhB solution (5 mg L^−1^). Then, the suspension was stirred for 30 min in the dark to ensure the establishment of an adsorption/desorption equilibrium. During the NIR irradiation, 3 mL reaction solution was taken and centrifuged every half an hour, and the filtrate was measured on a UV-Visible spectrometer at a maximum absorption wavelength of 554 nm to determine the concentration of RhB.

### 2.7. Characterizations

The sizes and morphologies of all samples were observed by scanning electron microscopy (SEM, JSM-6700F, JEOL, Tokyo, Japan) and transmission electron microscopy (TEM, JEM-2011, JEOL, Tokyo, Japan). High resolution electron microscopy (JEM-2011, JEOL, Tokyo, Japan), scanning transmission electron microscopy-high-angle annular dark field images (STEM-HAADF, JEM-2011, JEOL, Tokyo, Japan) and electron dispersive spectroscopy (EDS) mapping were conducted using the TEM (JEM-2011, JEOL, Tokyo, Japan). The crystallinity and phase structure of the samples were characterized by X-ray diffraction (XRD) with the Cu-kα (λ = 1.5406 Å) radiation (Rigaku, Japan). Fourier transform infrared (FTIR) absorption spectrawere obtainedusing a Nicolet 8700 system (Thermo Scientific, Waltham, MA, USA). The X-ray photoelectron spectroscopy (XPS) measurements were carried out on an ESCALAB 250 system with a monochromatic Al Kα X-ray source (Thermo-VG Scientific, West Sussex, UK). Ultraviolet-visible-near-infrared (UV-Vis-NIR) diffuse reflectance spectra were recorded by a Shimadzu SolidSpec-3700 system (Tokyo, Japan) equipped with an integrating sphere, and BaSO_4_ was used as the reference. The upconversion emission spectra excited at 980 nm were measured on a JY Fluorolog-3-Tou luminescence spectrometer. Magnetic properties of the samples were characterized using a physical property measurement system (PPMS DynaCool, Quantum Design, San Diego, CA, USA). 

## 3. Results and Discussion

**Figure 1:**
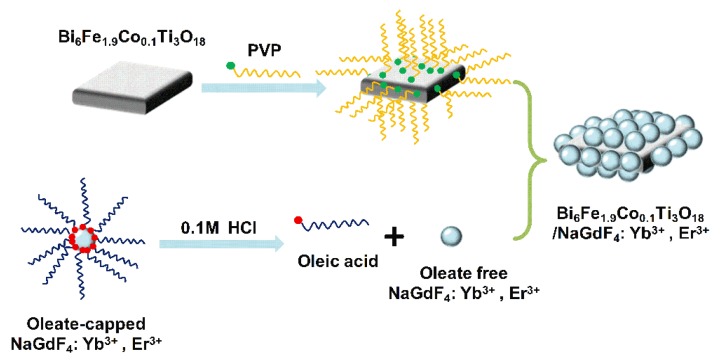
The schematic representation of synthesizing Bi_6_Fe_1.9_Co_0.1_Ti_3_O_18_/NaGdF_4_:Yb^3+^, Er^3+^ (BFCTO/NGF) nanocomposites.

**Figure 2:**
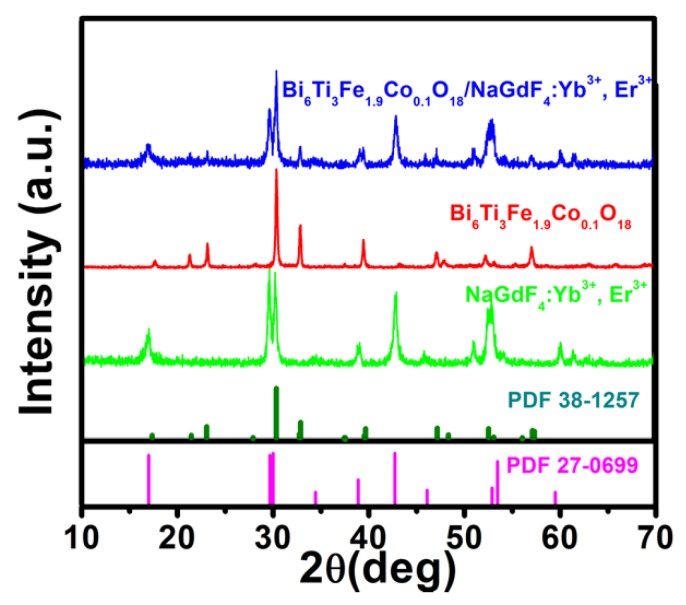
The X-ray diffraction (XRD) patterns of the NGF, BFCTO and BFCTO/NGF samples.

The detailed procedures of the BFCTO/NGF nanocomposites are outlined in Figure 1 and the Experimental Section. Figure 2 displays the XRD patterns of the NGF, BFCTO and BFCTO/NGF samples. Strong and sharp diffraction peaks were observed in all the samples, suggesting that the compounds are well-crystallized. The XRD pattern recorded in red suggests the formation of BFCTO pure phase, while all the XRD patterns indicated the characteristic BFCTO peaks with an orthorhombic structure (with a space group of *A*2_1_*am*, PDF 38-1257) [39]. The pattern recorded in pink reveals that the NGF hexagonal phase is obtained according to PDF 27-0699. The XRD pattern in blue displays both the characteristic peaks of the BFCTO and NGF phases, which suggests that NGF may be loaded onto BFCTO successfully. 

**Figure 3:**
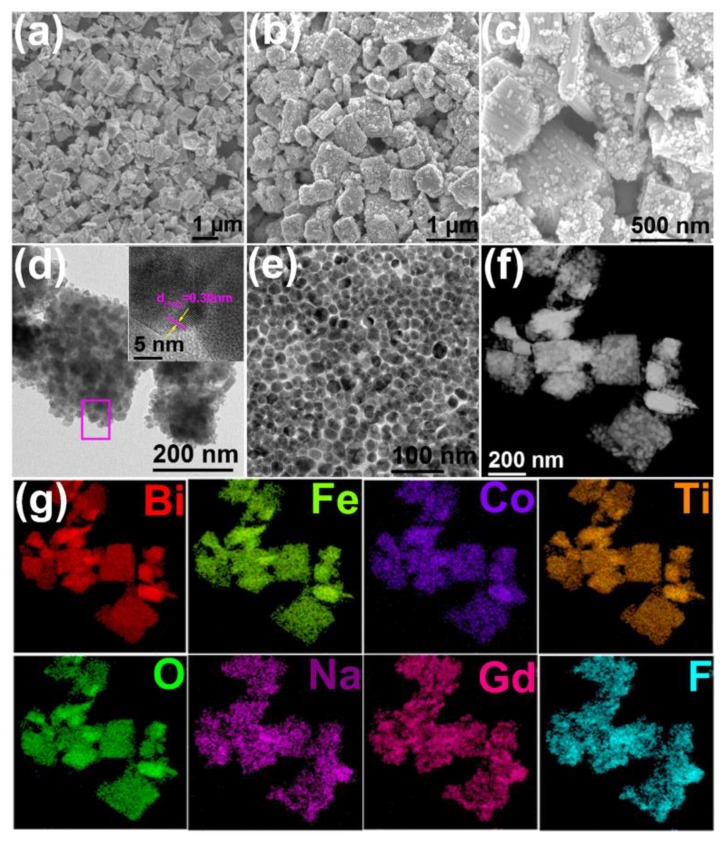
SEM images of (**a**) BFCTO-1.5 and (**b**,**c**) BFCTO-1.5/NGF nanoparticles; (**d**) TEM image of BFCTO-1.5/NGF nanoparticles (insert: the HRTEM image of NGF nanoparticle in magenta frame); (**e**) TEM image of NGF nanoparticles; (**f**) HADDF-STEM image of BFCTO-1.5/NGF; (**g**) the corresponding EDS elemental mapping of Bi, Fe, Co, Ti, O, Na, Gd and F elements.

The representative SEM images of the BFCTO-1.5 and BFCTO-1.5/NGF samples are shown in Figure 3a–c. BFCTO-1.5 nanoparticles are monodisperse and exhibit a nanoplate morphology, with a thickness and edge length of ~100 nm and ~1 μm, respectively (Figure 3a). There are two key factors for enhancing the catalytic activity of BFCTO by upconversion nanoparticles: (i) PVP surfactant could coordinate lanthanide ions through pyrrolidone groups on the surface of the NGF nanoparticles, which ensures the successful coating of NGF on the surface of BFCTO [40]; and (ii) the oleate ligand capped on the NGF nanoparticles is protonated by hydrochloric acid, thereby releasing oleic acid (OA) from the surface. This release of OA may increase the water solubility and reduce the fluorescence quenching of the NGF upconversion nanoparticles [41,42], which is beneficial for enhancing the NIR-responsive photocatalysis of BFCTO by upconversion nanoparticles. Figure 3b–d validates the successful loading of the NGF nanoparticles onto the BFCTO-1.5 nanoplates due to the modification of the PVP surfactant, with the size of the NGF nanoparticlesbeing ~30 nm (Figure 3e). Furthermore, the characteristic peaks of the oleate ligand disappear in the FTIR spectrum of the NGF nanoparticles, as shown Figure S1, suggesting that the NGF nanoparticles are oleic-free. HRTEM was also adopted to characterize the microstructures of the NGF nanoparticles, as shown in the insert of Figure 3d, and it indicated that the spacing between two adjacent lattice planes was 0.30 nm, fitting well with the (110) plane of the hexagonal phase of NGF (PDF 27-0699). To further verify the successful synthesis of the BFCTO-1.5/NGF nanocomposites, HADDF-STEM imaging and the EDS elemental mapping were conducted, as shown in Figure 3f,g. The HADDF-STEM image shows a sharp contrast between the NGF and BFCTO-1.50 nanoparticles, consistent with the TEM images in Figure 3b,c. The EDS elemental mapping in Figure 3g indicated that Na, Gd and F elements were distributed in the shell, while Bi, Fe, Co, Ti and O existed in the core, which further demonstrated the successful synthesis of BFCTO-1.50/NGF nanohybrids. 

**Figure 4:**
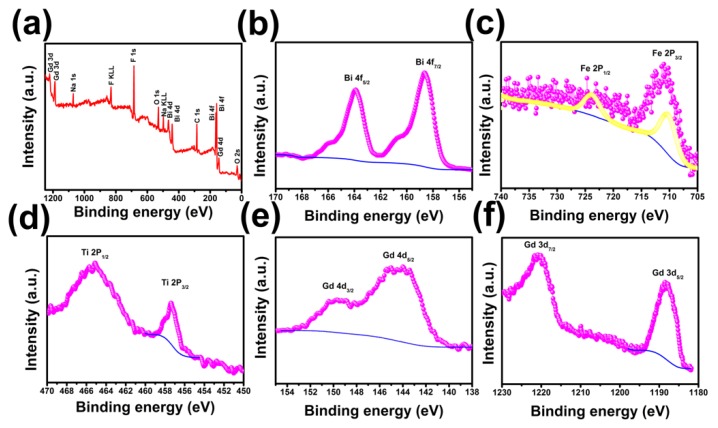
(**a**) The survey X-ray photoelectron spectroscopy (XPS) spectrum of the BFCTO-1.50/NGF nanocomposites; and high-resolution XPS spectra of (**b**) Bi 4f; (**c**) Fe 2p; (**d**) Ti 2p; (**e**) Gd 4d and (**f**) Gd 3d.

Additionally, XPS spectra were also conducted to determine the valence states of metal elements in the BFCTO/NGF nanohybrids (Figure 4). The bindingenergies obtained from the XPS analyses were corrected for specimen charging by referencing the C 1s line to 284.5 eV. The full survey spectrum revealed that the BFCTO-1.5/NGF nanohybrid was composed of Bi, Fe, Co, Ti, O, Na, Gd and F elements (Figure 4a). Figure 4b illustrates the core-level spectrum of Bi 4f in the nanohybrid, and the peaks located at 158.7 eV (Bi 4f_7/2_) and 164.0 eV (Bi 4f_5/2_) are referenced by Bi_2_O_3_ [43]. Figure 4c shows the Fe 2p core-level spectrum of the BFCTO-1.5/NGF nanohybrid, and the two peaks of Fe 2p at 723.81 and 710.2 eV—which match well with the spin-orbit split of Fe 2p_1/2_ and Fe 2p_3/2_, respectively [39]—are near to those in Fe_2_O_3_, suggesting that the Fe ions in the BFCTO/NGF nanocomposite have +3 valence states. The binding energies at 465.15 and 457.26 eV correspond to the binding energy of Ti 2p_1/2_ and Ti 2p_3/2_ (Figure 4d), implying that the doped Ti ions in the BFCTO/NGF nanohybrid have +4 valence states [44]. Additionally, Figure 4e shows the Gd 4d_3/2_ peak at 149.7 eV and the Gd 4d_5/2_ peak at 143.9 eV [45], and the Gd 3d_5/2_ peak at 1188.5 eV and Gd 3d_7/2_ peak at 1220.9 eV are also observed (Figure 4e) [46], which are characteristic of Gd^3+^ ions. Therefore, the XPS results indicate that the metal elements exist in the form of Bi^3+^, Fe^3+^, Ti^4+^ and Gd^3+^.

**Figure 5:**
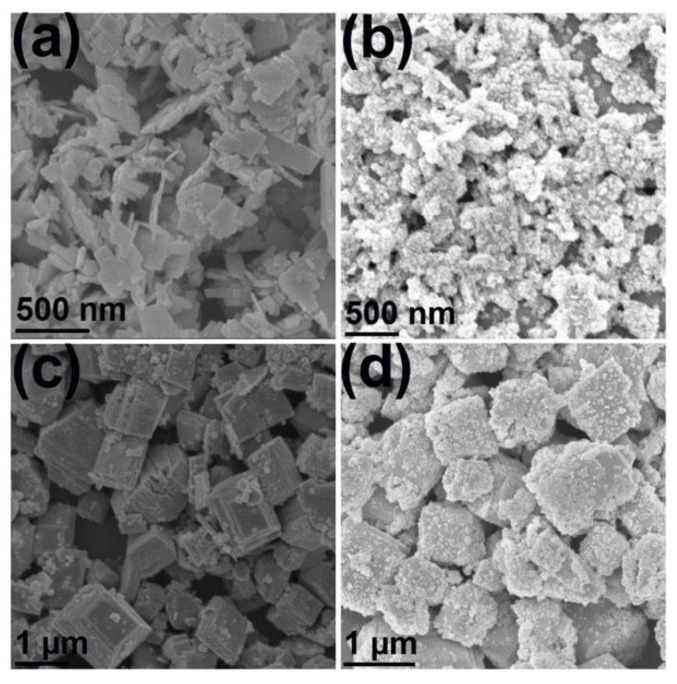
The SEM images of (**a**) BFCTO-1.0; (**b**) BFCTO-1.0/NGF; (**c**) BFCTO-2.0 and (**d**) BFCTO-2.0/NGF samples.

The SEM images of BFCTO-1.0, BFCTO-1.0/NGF, BFCTO-2.0 and BFCTO-2.0/NGF samples are shown in Figure 5. The BFCTO-1.0 sample displays a nanosheet-like morphology, with a thickness and edge length of ~40 nm and ~1 μm, respectively (Figure 5a). The BFCTO-2.0 sample exhibits a well-defined shape of a truncated tetragonal bipyramid, the thickness and edge length of which ranges from 100 nm to 1 μm and 1 to 2 μm, respectively (Figure 5c) [26]. Evidently, the coating of NGF did not change the size and morphology of BFCTO because the NGF nanoparticles were just absorbed onto the BFCTO nanoparticles’ surfaces through the amide bond in PVP.

**Figure 6:**
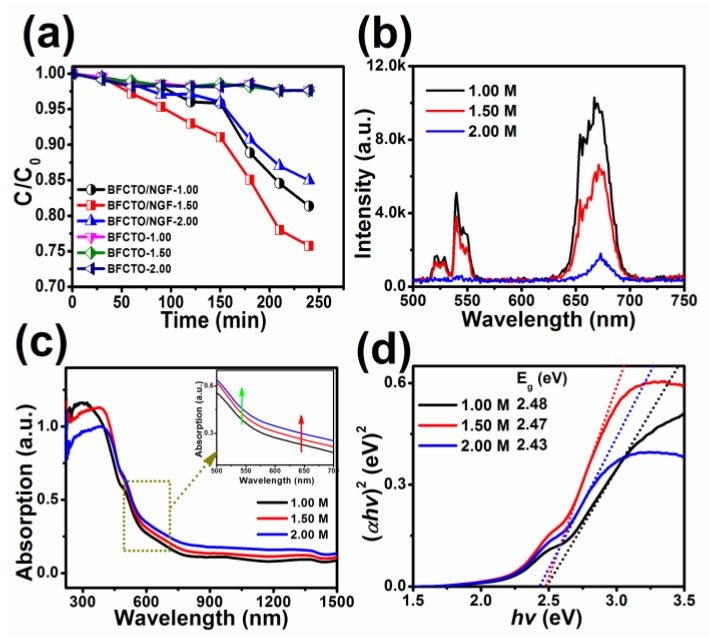
(**a**) The irradiation time dependence of Rhodamine B (RhB) degradation in various BFCTO and BFCTO/NGF aqueous dispersions under near-infrared (NIR) irradiation of 980 nm, with a power of 1.0 A; (**b**) the upconversion emission spectra of BFCTO/NGF nanoparticles excited at 980 nm; (**c**) the ultraviolet-visible-near-infrared (UV-Vis-NIR) diffuse reflectance spectra of BFCTO/NGF nanoparticles (insert: the absorption magnification of yellow dot area); (**d**) the relationship between (*αhv*)^2^ and (*hv*) photon energy of all BFCTO/NGF nanoparticles (insert: Corresponding E_g_ values).

After successfully synthesizing the BFCTO/NGF nanoparticles, we were in a position to evaluate the corresponding photocatalytic activities. To identify the dependence of morphology on photocatalysis performance, the degradation rate of all the samples was normalized by the specific surface area, which is 17.733, 11.731 and 11.610 m^2^ g^−1^ for BFCTO-1.0/NGF, BFCTO-1.5/NGF and BFCTO-2.0/NGF, respectively. Figure 6a displays the irradiation time dependence of RhB degradations in various BFCTO and BFCTO/NGF aqueous dispersions under NIR irradiation of 980 nm, with a power of 1.0 A. Evidently, all the BFCTO samples exhibited no activity after the adsorption/desorption equilibrium. In contrast, with the loading of NGF upconversion nanoparticles, NIR-responsive photocatalysis activity was observed in all the BFCTO/NGF samples, andBFCTO-1.5/NGF degrades 25% of RhB after 240 min irradiation at 980 nm, better than theBFCTO-1.0/NGF and BFCTO-2.0/NGF samples. 

To explain the effect of NGF-loading on BFCTO nanoparticles with different morphologies, the upconversion emission spectra of all the BFCTO/NGF samples were measured (Figure 6b). Under the excitation of a 980 nm continuous wave diode laser, the green emission centered at 543 nm and red emission centered at 658 nm were observed, originating from the transitions ^2^H_11/2_/^4^S_3/2_→^4^I_15/2_ and ^4^F_9/2_→^4^I_15/2_, respectively, for the Er^3+^ ions, indicating that the loading of NGF nanoparticles could extend the absorption of BFCTO from Vis to NIR light. All of the BFCTO/NGF samples exhibited stronger red emission than green emission due to high Yb^3+^ doping concentration (20 at%), and stronger absorption of BFCTO in the green band (Figure 6c). Furthermore, the intensity of the upconversion emissions decreased in the order of I_BFCTO-2.0/NGF_ < I_BFCTO-1.5/NGF_ < I_BFCTO-1.0/NGF_ (Figure 6b), suggesting that absorption in the NIR region increases in the order of I_BFCTO-2.0/NGF_ > I_BFCTO-1.5/NGF_ > I_BFCTO-1.0/NGF_, consistent with the results in Figure 6c. Furthermore, upconversion nanoparticles may reduce the electrical conductivity of BFCTO photocatalysts due to NGF’s electrical insulating properties. The UV-Vis-NIR diffuse reflectance spectra in the wavelength range of 250–1500 nm for all the BFCTO/NGF samples, displayed in Figure 6c, are relevant to the electronic structure, and are the key factors to determine the band gaps. According to the UV-Vis-NIR diffuse reflectance spectra in Figure 6c, the band gap values are calculated by the following equation: *αhv* = A (*hv* − E_g_)^n/2^, where *α*, *hv*, A, and E_g_ represent the absorption coefficient, photon energy, proportionality constant and gap band, respectively. BFCTO possesses a direct electron transition, therein n = 1. Figure 6d shows the plot of the (αhv)^2^ verse E_g_, from which the band gaps of the BFCTO/NGF samples were estimated by extrapolating a straight line to the abscissa axis. The band gaps were found to be 2.48, 2.47 and 2.43 eV for BFCTO-1.0/NGF, BFCTO-1.5/NGF and BFCTO-2.0/NGF, respectively. The intrinsic absorption edge of BFCTO-2.0/NGF has an obvious red-shift by 10 nm to that of BFCTO-1.0/NGF.

**Figure 7:**
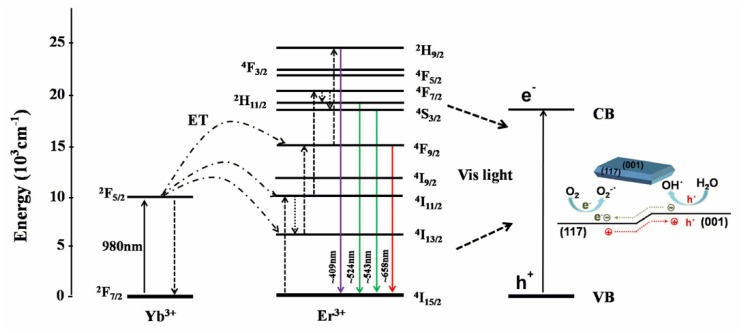
Energy level diagram and the energy transfer between Yb^3+^ and Er^3+^ showing the proposed upconversion mechanism under 980 nm excitation. The photoexcitation of BFCTO by the upconversion emission in the Vis region is also shown.

The photodegradation mechanism of BFCTO/NGF under 980 nm irradiation is shown in Figure 7. In the upconversion luminescent NGF nanoparticle, the excited Yb^3+^ ions act as sensitizers to absorb the 980 nm photons, and then transfer this energy to Er^3+^ ions to emit Vis light. The emission peaks centered at 524, 543, and 658 nm for NGF are attributed to the Er^3+^ transitions of ^2^H_11/2_→^4^I_15/2_, ^4^S_3/2_→^4^I_15/2_, and ^4^F_9/2_→^4^I_15/2_, respectively. Meanwhile, these emission peaks are in the Vis range, which are fully in the BFCTO absorption range, leading to photoexcitation of the BFCTO nanoparticles in BFCTO/NGF. Therefore, the holes (h^+^) on the valence band (VB) and the electrons (e^−^) on the conduction band (CB) were able to be generated to decompose the RhB molecules. The oxidative holes could react with H_2_O to produce hydroxyl radicals (∙OH), while in the reduction reaction the electrons reacted with O_2_ to form superoxide radicals (O_2_^−^) [47]. Furthermore, in previous work, we indicated that the BFCTO-1.50 sample showed the highest photocatalysis efficiency, which may have been due to the nanoplates in the BFCTO-1.50 sample having the appropriate area ratio between the {001} facets and the {117} facets. This resulted in a similar reaction ratio on these two facets, thereby efficiently inhibiting the recombination of the charge carrier [23]. Consequently, we predicted that the BFCTO-1.5/NGF would show the best photocatalytic activity, which could be attributed to the effect of {001} and {117} crystal facets with the optimal ratio.

**Figure 8:**
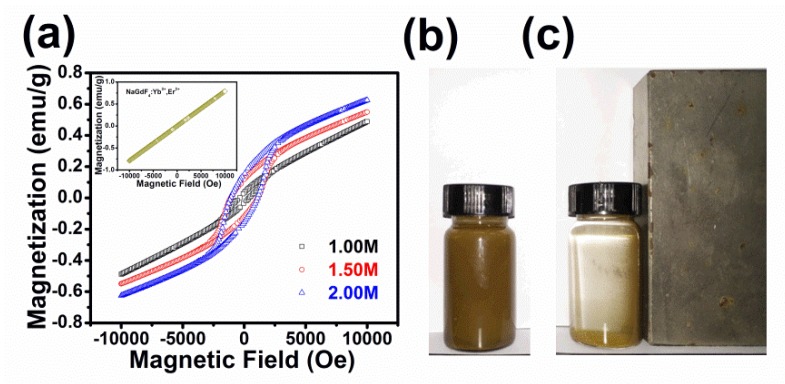
(**a**) Magnetization (M)-applied magnetic field (H) hysteresis loops of the BFCTO/NGF samples (insert: M-H loop of NGF); and the photos of BFCTO-1.0/NGF solution; (**b**) without and (**c**) with a magnet of 0.1 T.

The magnetic properties of all the BFCTO/NGF nanocomposites at room temperature were also measured to investigate their potential as a magnetic recyclable catalyst. The magnetization hysteresis (M-H) loops are shown in Figure 8a. It can be seen that all the samples exhibit spontaneous magnetic moments, indicating their ferromagnetic nature. Among the three samples, BFCTO-2.0/NGF exhibited a maximum remnant magnetism (2*M*_r_) of ~0.32 emu/g, and the coercive field 2*H*_c_ of BFCTO-2.0/NGF is ~1092 Oe. Due to the paramagnetic properties of the NGF nanoparticles (insert of Figure 8a), the BFCTO/NGF samples showed unsaturated magnetization when a high magnetic field was applied. Additionally, Figure 8b,c displays the photos of BFCTO-1.0/NGF solution without and with a nearby magnet of 0.1 T, which indicates that the BFCTO/NGF samples have the potential as novel, recyclable and efficient NIR light-driven photocatalysts.

## 4. Conclusions

In summary, the BFCTO/NGF nanocomposites were successfully synthesized by the hydrothermal method followed by an assembly method, wherein the size and morphology of the BFCTO component was well-adjusted by changing the concentration of NaOH. Under the NIR irradiation of 980 nm by a continuous wave diode laser, all the BFCTO samples exhibited no activity. In contrast, with the loading of the NGF upconversion nanoparticles, high photocatalytic activity was observed in the BFCTO/NGF samples, indicating that the loading of NGF nanoparticles could extend the absorption of BFCTO from Vis to NIRlight. Among the three samples, BFCTO-1.5/NGF displayed the best NIR-responsive photocatalysis activity, which could be attributed to the effect of {001} and {117} crystal facets with the optimal ratio. Furthermore, the ferromagnetic property of the BFCTO/NGF sample indicates itspotential as anovel, recyclable and efficient NIR light-driven photocatalyst.

## Supplementary Materials

The following are available online at http://www.mdpi.com/2079-4991/8/7/534/s1, Figure S1: The FT-IR spectra of oleic acid, oleic-capped NGF and oleic-free NGF nanoparticles.
