# Supplementary material for: Extended Near-Infrared Photoactivity of Bi6Fe1.9Co0.1Ti3O18 by Upconversion Nanoparticles
Source: Nanomaterials (Basel). 2018 Jul 16;8(7):534. doi: 10.3390/nano8070534 (PMC6071231; doi:10.3390/nano8070534)
Supplement: Supplementary file 1 [file nanomaterials-08-00534-s001.pdf]

Supplementary Materials

Extended Near-Infrared Photoactivity of $Bi_6Fe_{1.9}Co_{0.1}Ti_3O_{18}$ by Upconversion Nanoparticles

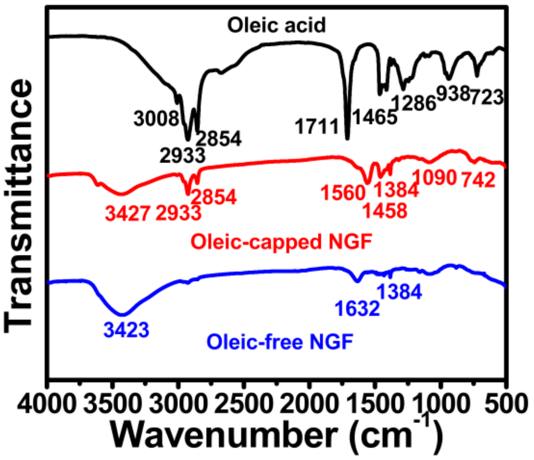

Figure S1. The FT-IR spectra of oleic acid, oleic-capped NGF and oleic-free NGF nanoparticles.
